# Wearable Systems for Home Monitoring Healthcare: The Photoplethysmography Success Pros and Cons

**DOI:** 10.3390/bios12100861

**Published:** 2022-10-12

**Authors:** Antonio Lanata

**Affiliations:** Department of Information Engineering, University of Florence, 50139 Firenze, Italy; antonio.lanata@unifi.it

**Keywords:** wearable systems, photoplethysmography, continuous monitoring, cardiovascular disease, movement artifacts

## Abstract

The widespread use of remote technology has moved medical care services into individuals’ homes. In this perspective, the ubiquitous computing research proposes self-management and remote monitoring to help patients with healthcare in low-cost everyday home usage systems based on the latest technological advances in sensors, communication, and portability. This work analyzes recent publications on the paradigm of continuous monitoring through wearable and portable systems, focusing on photoplethysmography (PPG) advances and referencing the current systematic study proposed by Fine et al. The study revised the literature highlighting the pros and cons of using the PPG system for fitness, wellbeing, and medical devices. However, future works should focus on the standardization of the practical use and assessment of the quality of the PPGs’ output. For clinical parameter extraction methodology in terms of biological sites of application and signal processing methods, PPG is the most convenient and widely used system potentially suitable for the decentralized paradigm of continuous monitoring healthcare concepts.

In the last decades, in most countries of the world, the structure of the healthcare system has relied on centralized hospitalization, in which ill citizens move to a clinic to receive a medical diagnosis. While this model has been effective in the past, it is now insufficient in offering the appropriate care services due to several challenges derived from global epidemics and the ability to manage dwindling resources [1]. Nowadays, we are experimenting with the widespread use of remote technology to move medical care services locally into individuals’ homes. In this perspective, the ubiquitous computing research findings aim at helping patients with healthcare self-management and remote monitoring, providing low-cost everyday home usage systems based on the latest technological advances in sensors, communication, and portability.

Mobile, wearable sensing technology (MWST) allows for the monitoring of physical and social behavior, either individually or collectively. Wearables can offer a convenient approach to gathering data in naturalistic conditions [2]. MWST enables long-term and continuous patient health monitoring, supporting early disease detection and independent living, enhancing rehabilitation treatment, and assessing wellbeing [3].

The article presented by Fine et al. reports on a comprehensive investigation of the technical and theoretical applications of Photoplethysmography (PPG) [4], which is currently one of the most widely used wearable systems, to investigate and monitor cardiovascular activity. PPG represents a low-cost optical technique for evaluating changes in blood volume within tissues, resulting in a simple waveform that does not require a high-level signal processing methodology for extracting health information such as Heart Rate (HR). The PPG output can be split into a quasi-static (DC) and a pulsatile component (AC). Moreover, when it is used as a medical device, more complex analysis is needed for the robust and reliable measurement of clinical parameters. The PPG could yield valuable clinical data on heart status, blood pressure, respiratory activity, sympathovagal balance, and heart rate variability.

However, as reported in this editorial, wearable monitoring tools for clinical application go beyond fitness and heart rate evaluation, and noise sources that originate from individual patient variations (e.g., skin tone, obesity, age, and gender), physiology (e.g., respiration, venous pulsation, body site of measurement, and body temperature), and external perturbations of the device itself (e.g., motion artifact, ambient light, and applied pressure to the skin) need to be considered.

The first part of the work concerns the technical overview of the PPG concept and how its signal outcome is obtained, specifying some interesting fiducial waveform parts on which quantitative parameters can be extracted. Moreover, it carefully describes PPG’s clinical implications in cardiovascular disease (CVD) monitoring, highlighting the current gap in clinical practice between prevention and diagnosis in CVD assessment. The PPG system used at home for long-term monitoring can fill the gap. One crucial aspect underlined by the article is the relation between commercial PPG products and governmental associations’ approval, such as the US Food and Drug Administration (FDA). Nowadays, PPG systems are mainly approved for nonclinical use (i.e., measurement of Heart Rate for fitness). The only examples approved are those PPG systems used together with a specific software suite. Fine and his colleagues state that this is due to the inaccuracy of information that is mainly related to the high number of noise sources affecting the PPG parameter extraction. In regard to this concern, Fine et al. widely span the current literature that offers a comprehensive vision of the noise sources and how they affect the PPG waveform and its derivative signals, maintaining the aim of providing relevant indications for PPG system design. 

The analysis of noise sources is then articulated in three main sections: individual variation in the population, physiology, and external factors.

Regarding the first source of noise, the work reports four main factors: skin tone, obesity, age, and gender. All these factors affect the quality and morphology of the PPG signals. Skin tone, evaluated by many authors through the Fitzpatrick scale [5], strongly affects the AC PPG signal due to the different absorption of eumelanin from tissues having a direct effect on the Signals-to-Noise Ratio (SNR). As reported by many authors, a higher level of pigmented skin correlates with a lower value in SNR [6,7]. Even if most reported works have highlighted some different quantitative results, for all experimental protocol conditions, e.g., resting state or physical exercises, they have a relationship between PPG signal and both the wavelengths of light in common, due to the light–tissue interaction, and the relative distance between the light source and detector exists. The editorial underlines the importance of future studies to quantify these two crucial aspects. Obesity, a factor often evaluated in terms of body mass index (BMI), is an accumulation of body fat, provoking changes in skin thickness, blood flow, and oxygen saturation. It has a direct effect on the BC component of PPG. It can be observed that there is a direct correlation between BMI, blood flow and skin thickness, while there is an inverse correlation between BMI, capillarity density and oxygen saturation. 

As seen for obesity, age affects the PPG value and its use for cardiovascular health assessments. This is mainly due to the anatomical and physiological changes in vascularization (e.g., the tunica intima and tunica media layers increase the amount and density of collagen) and skin thickness. It is fascinating to observe that one of the main influences of chronological age is related to second derivative PPG and acceleration plethysmography (APG). The work reported correlations involving the APG, attributing a positive direct relationship between PPG amplitude and age to capillary depletion due to similar skin thicknesses observed between older subjects and children. Overall, the impact of age-related skin thickness changes on PPG may depend on the body site of measurement. However, it can be used in modeling blood pressure from PPG.

The physiological differences between men and women can be found in the PPG waveform. The physiological baseline differences can skew PPG signals for cardiovascular health per gender detection. The article shows how men have greater low-frequency signal components whilst women have more significant high-frequency components. Some experiments on commercial devices such as Fitbit, Apple Watch, Microsoft Band, Samsung Gear, and the Basis Peak watch showed a significantly higher device measurement error in males than females for all devices [8].

The second influencing item covered in this study concerns how physiological mechanisms can affect the baseline values or periodicity of the PPG waveform or even change the waveform shape [9]. Specifically, four mechanisms are considered: respiration, venous pulsations, body site measurement, and local body temperature distribution. Respiration mainly modulates the baseline and amplitude of the PPG signal. Specifically, shifts in blood volume due to changes in respiratory activity will cause a corresponding change to the baseline amplitude of the PPG signal [10]. Interestingly, it is also suggested that the coupling between the respiratory and the autonomic nervous system provides a mechanical effect on the vascular system detectable by the PPG. Due to the reported influences, it is interesting to observe that several signal processing techniques have been developed to extract respiration information from the PPG signal directly. The venous system similarly contributes periodicity to the PPG signal, which originates from the vascular network of tiny vessels transporting deoxygenated blood from the capillaries to the heart. Continuous monitoring of both venous and artery contributions to the PPG waveform indicates a strong correlation between the diastolic peak in the plethysmograph and peaks in the venous pulse at the peripheral locations. Moreover, the noninvasive venous pressure measurement can help diagnose clinically relevant conditions such as congestive heart failure or valvular heart disease [11,12].

In recent years, we have seen a wide number of PPG tools developed for different body site locations such as the fingers, wrist, brachia (upper arm), earlobe, ear cartilage, superior auricular region, esophageal region, and the forehead, highlighting the relevant influences that the body site measurements have on the PPG signal. Major problems arise when the processing techniques to assess cardiovascular risk are applied without considering the variability of anatomy location, such as skin thickness and basal blood perfusion. Overall, Fine et al. demonstrated that different anatomies, skin thickness, and blood perfusion play a role in the strength of the quality of the PPG signal. Moreover, as a result, the finger is likely to be the most successful anatomy site to be used.

Another essential physiological aspect is thermoregulation, fundamental in homeostatic function. Significant body temperature variations in response to external stimulation can affect PPG waveform amplitude and even the time between waveforms. However, many studies also suggested that external temperature has a much more significant effect on the PPG waveform.

The last relevant factor of influence is composed of many elements that Fine et al. grouped in a general “external factor” section. This attractive section focuses on how motion artifacts, ambient light, and applied pressure to the measurement site can affect the PPG waveform.

Body movements, e.g., physical exercises such as walking or jogging, cause significant fluctuations (i.e., movement artifact (MA)) in the PPG signal within a frequency range of 0.1 and 20 Hz, which is in the same bandwidth of heart rate and other PPG derived parameters. With the increase in market requests for heart rate monitoring devices, there is an increased need for motion artifact identification and elimination. The study highlighted how the PPG systems based on green light have the lowest motion artifact noise. Moreover, recently, we have seen an increased development of systems that integrate additional sensors for detecting the type and intensity of the movements to implement directly on-board different kinds of signal processing tools (e.g., adaptative filtering) for removing MA from PPG signals. Some others implemented several statistical or time-based signal processing tools for the same scope without additional sensors [13].

On the contrary, ambient light has a significant effect on the PPG signal, as reported by Fine et al., since PPG is mainly constituted of a light source and a light detector, and the latter has a sensibility greater than the light source. It is therefore sensible to other sources of light coming from the external ambient. Generally, ambient light interference is found both at 0 Hz, such as sunlight, or a variable frequency with a magnitude larger than the pulsatile (AC) PPG component of the body, which leads to a saturation of the sensors. Over the years, many strategies have been adopted to protect the PPG system from this noise source, for example, employment of an opaque medium at the base, encasement of the active area of the photodetector in a red plastic to filter out ambient light, and the addition of a foam between the source and detector to prevent direct coupling from them. Fine et al. summarized that the accuracy of PPG measurements is limited by environment noise interference that can be compensated by various methods such as optical shielding of the transducer and selective filtering of noise outside the PPG bandwidth. Moreover, signal processing methods can be integrated into the signal amplifier to reject DC photocurrent in PPG sensors.

Finally, changes in contact force affect several characteristics of the PPG waveform, such as AC amplitude, DC offset amplitude, AC/DC amplitudes ratio, and the normalized pulse area. The modification in these fundamental features can affect other measurements, such as the second derivative of a PPG signal, the relationship between the frequency response of a PPG signal and a blood pressure signal, or the pulse transit time (PTT), which is used as an indicator for fluctuating stiffness or elasticity in arteries and blood pressure. This noise could be mitigated by integrating sensors measuring the applied force to standardize PPG measurements not to skew the signal.

In summary, this editorial focuses on technical and practical concepts related to remote and continuous PPG devices, primarily when they are intended for clinical applications. The study clearly shows the number of noise sources involved in the design of such a system. Fine et al. collected and evaluated these noise sources through published works and used knowledge about photoplethysmography and light/tissue interactions to summarize the results and provide guidance for future PPG-based devices. An interesting point of view is the division of noises into three categories: individual variations, physiological processes, and external perturbations. Results showed that even if potential solutions have been documented for many noise sources, only a few have found a complete solution. Future research in defining true regulated health monitoring PPG devices should include more extensive studies considering all the noise sources in different populations.
